# Treatment Strategies for Primary Root Fractures: A Case Series

**DOI:** 10.7759/cureus.103288

**Published:** 2026-02-09

**Authors:** Mridula Goswami, Akansha Gupta, Rimshheanam Rimshheanam

**Affiliations:** 1 Pediatric and Preventive Dentistry, Maulana Azad Institute of Dental Sciences, New Delhi, IND

**Keywords:** pediatric and preventive dentistry, primary anterior teeth, root fracture, traumatic dental injury, young child

## Abstract

Traumatic dental injuries (TDIs) constitute a clinically significant concern in pediatric dentistry, necessitating special attention to the trauma affecting primary dentition due to their potential to cause damage to the underlying permanent successors. The anatomical proximity of primary tooth roots to developing permanent tooth germs increases the risk of sequelae, making root fractures in primary teeth more challenging to diagnose and manage. This case series highlights the diagnosis and management of TDIs in three pediatric patients aged 4-7 years, each presenting with varying severities of horizontal root fractures and associated symptoms in the primary maxillary incisors.

Among traumatic dental injuries in children, root fractures of primary teeth are clinically significant, with horizontal root fractures being the most commonly reported type, and their prognosis is largely determined by the fracture location and extent. Clinical presentations in this case series ranged from no mobility and the displacement of the teeth to severe luxation injuries. Management strategies were modified according to each case to have a favorable prognosis followed by esthetic rehabilitation. All patients were monitored through clinical and radiographic follow-ups, demonstrating favorable healing outcomes with no evidence of any adverse sequelae. The management of horizontal root fractures in primary teeth requires a case-specific approach based on the fracture level, tooth mobility, the root status of the primary tooth, and potential impact on the permanent successors.

Each case is unique; hence, early diagnosis, appropriate intervention, and regular follow-ups are essential to achieve favorable outcomes in root fractures of primary teeth and to safeguard the developing permanent dentition.

## Introduction

Traumatic dental injuries (TDIs) refer to injuries affecting the teeth and their supporting structures as a result of external force, including fractures, luxation injuries, and avulsion. TDIs are common in children and adolescents, with nearly 50% of children experiencing a dental injury by the age of 18 [1,2]. Trauma to the primary dentition is particularly prevalent in early childhood, primarily attributed to a lack of motor coordination and increased susceptibility to falls during this developmental stage. The global prevalence of TDIs in primary teeth was reported to be 22.7%, whereas the overall prevalence of TDIs in primary teeth in India was 24.2% [3-5]. Etiology for TDIs in primary teeth in India includes falls (43%), followed by sports-related injuries (26%), collisions (12%), road traffic accidents (8%), violence (7%), and other causes (7%) [6]. Indoor falls account for the highest prevalence (52%) among traumatic incidents, exceeding those occurring outdoors or on stairs, and are most frequently associated with play-related activities within the home or during recreational and sporting events [7,8].

The maxillary central incisors are the most frequently affected teeth in TDI, likely due to their prominent position in the dental arch, with primary maxillary central incisors accounting for 73.9% of cases [4]. The most prevalent type of TDI in primary teeth includes luxation injuries (41.8%), followed by avulsion (19.4%) and then root fractures and crown fractures each accounting for 5.8%, concussion at 3.7%, and isolated fractures being the least common at 2.9%. Root fractures in primary teeth affect the dentin, cementum, periodontal ligament, and pulp [9]. These fractures occur due to severe traumatic impact to the crown near the gingiva or in correspondence to the alveolar bone [3]. Though the prevalence of root fractures in the primary teeth is low, they present considerable diagnostic and therapeutic challenges and are often complicated by other surrounding injuries.

Root fractures can be broadly classified based on the direction of the fracture line in relation to the long axis of the tooth as horizontal (perpendicular), oblique (at an angle), or vertical (parallel). Transverse or horizontal root fractures can be further divided into different categories based on the location, extent, and number of fracture lines and the position of the coronal fragment [10].

Trauma to primary teeth may result in complications such as pulp necrosis, root resorption, crown discoloration, and premature tooth loss, leading to pain, infection, and functional impairment [11]. Given the close anatomical proximity to developing permanent tooth buds, such injuries can also cause long-term sequelae, including enamel hypoplasia, discoloration, root dilaceration, and eruption disturbances [12]. These potential outcomes highlight the critical need for early diagnosis, appropriate management, and long-term clinical and radiographic monitoring to preserve the health of both primary and permanent dentitions, as emphasized by the 2020 guidelines of the International Association of Dental Traumatology (IADT) for the management of primary tooth trauma [7].

Clinical and radiographic follow-ups are essential to assess healing and monitor for any unfavorable outcomes. The present case series is a compilation of three pediatric cases highlighting the diagnosis and individualized management of traumatic root fractures in primary maxillary incisors, with various clinical and radiographic outcomes.

## Case presentation

**Figure 1:**
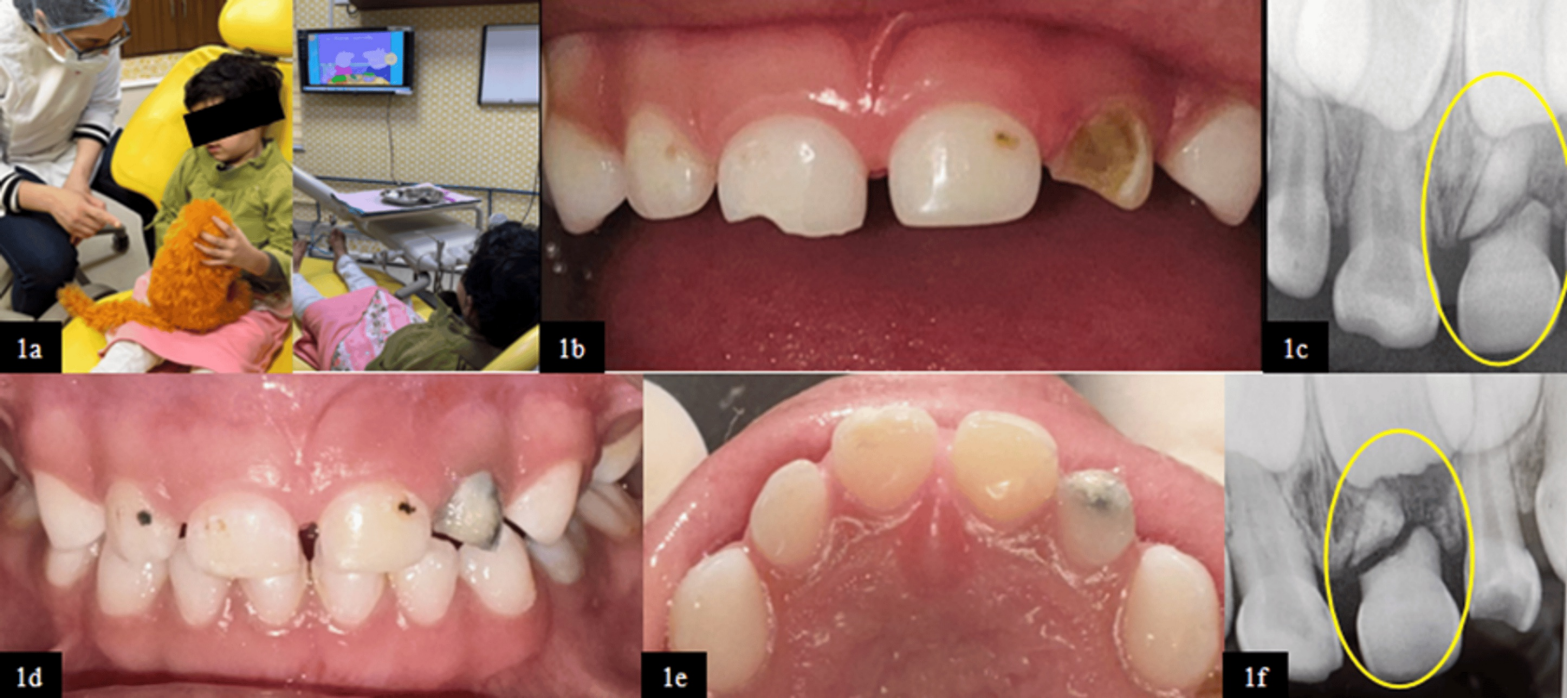
(1a) Behavior shaping of a child using audiovisual distraction. (1b) Frontal view showing enamel-dentin fracture wrt 51. (1c) Pre-operative RVG wherein 61 shows cervical root fracture. (1d) Frontal view at two-year follow-up. (1e) Maxillary occlusal view at two-year follow-up. (1f) RVG wrt 51 and 61 at two-year follow-up RVG, radiovisiography; wrt, with respect to

The present case series depicts three cases of horizontal root fractures in primary maxillary anterior teeth in pediatric patients within the age group of 4-7 years. The patients reported to the department of pediatric and preventive dentistry with different chief complaints. Guardians of all the patients gave a history of trauma due to a fall with no history of loss of consciousness, vomiting, seizure, and ear, nose, or throat bleed. A comprehensive medical and family history and previous dental history, with general physical, clinical, and radiographic examination, were collected for each case. Non-pharmacological behavior management techniques such as communication, tell-show-do, and modelling were implied to achieve cooperation (Figure 1a). After obtaining informed consent from the parent/guardian and explaining the treatment plan, emergency care was provided, followed by appropriate functional and esthetic dental rehabilitation. As per the 2020 IADT guidelines, periodic clinical and radiographic follow-ups were done [7].

Case 1

A four-year-old female patient reported with a complaint of a chipped upper right front tooth due to a fall two months prior.

On clinical examination, an uncomplicated crown fracture involving enamel and dentin in the primary maxillary right central incisor (tooth 51) was diagnosed (Figure 1b). It was only after radiovisiographic (RVG) evaluation that the trauma to tooth 61 was identified to be more severe than clinically apparent, revealing an oblique root fracture extending from the cervix to the middle third of the root, characterized by a malaligned fracture line despite the absence of clinical mobility or occlusal interference (Figure 1c).

After clinical and radiographic evaluation, the restoration of tooth 51 was done using a type II glass ionomer cement (3M ESPE Ketac Molar Glass Ionomer Restorative Material, Bangalore, India). Conservative management involving periodic follow-ups was adopted for tooth 61 due to the absence of any pathology. Two-year follow-up radiograph showed the stable positioning of both root and crown fragments and the absence of pathological root resorption with no periapical pathology, indicating favorable healing (Figure 1d-1f). Additionally, the normal eruption of the permanent successor tooth 21 and the physiological resorption of the apical fragment of tooth 61 were observed, demonstrating favorable healing and the normal developmental progression of the permanent tooth without any disturbance in its eruption pathway.

Case 2

A seven-year-old male patient reported with a complaint of discoloration in the upper right front tooth. Detailed history revealed a traumatic incident five months prior for which no dental intervention was sought at the time, as reported by the parent.

**Figure 2:**
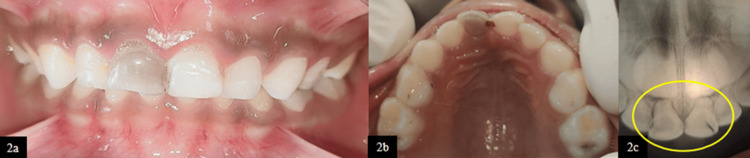
(2a) Frontal view showing discoloration wrt 51. (2b) Maxillary occlusal view. (2c) Pre-operative IOPA wrt 51 and 61 IOPA, intraoral periapical radiography; wrt, with respect to

**Figure 3:**
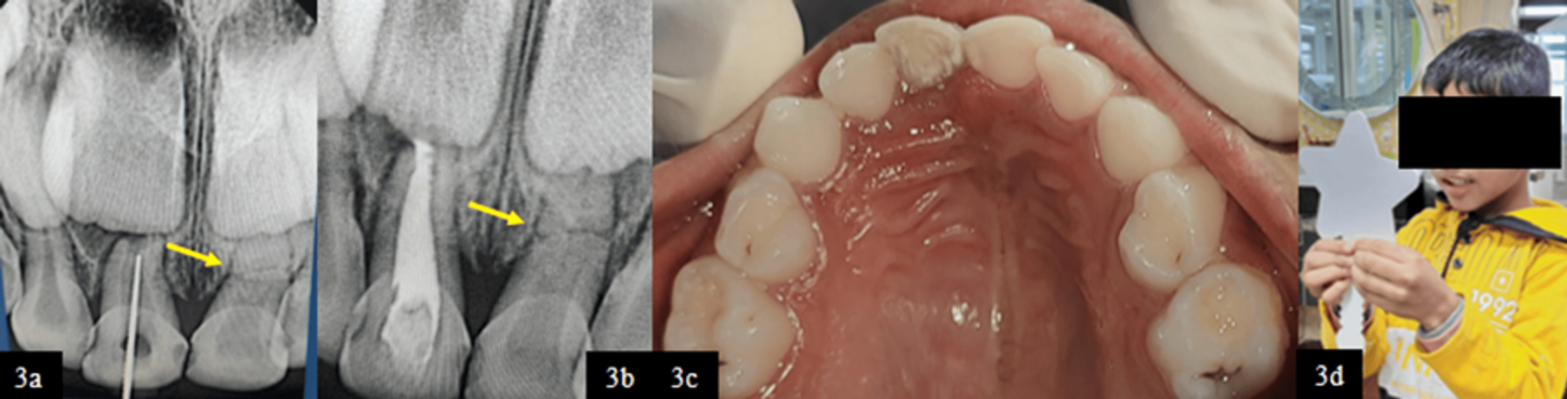
(3a) RVG showing working length wrt 51 and root fracture identified at the middle third wrt 61. (3b) RVG showing Metapex obturation wrt 51. (3c) Post-operative occlusal view. (3d) Post-operative extraoral view demonstrating a satisfied patient following the completion of treatment RVG, radiovisiography; wrt, with respect to

Clinical examination revealed an Ellis class IX fracture with grayish-black discoloration in tooth 51 (Figure 2a, 2b). Intraoral periapical radiography (IOPA) demonstrated a periapical radiolucency associated with tooth 51, for which pulpectomy was planned (Figure 2c). During working length determination using RVG, a horizontal root fracture was incidentally detected in the middle third of the root of tooth 61 (Figure 3a). On clinical evaluation, the coronal fragment was intact, with no signs of displacement or mobility.

Tooth 61 was placed under observation as part of a conservative treatment strategy. Tooth 51 was esthetically rehabilitated with BioFlx crown (Kids-e-Dental BioFlx crown, Mumbai, India) after the completion of endodontic treatment by using Metapex (Meta Biomed Co. Ltd., Cheongju, South Korea) as the obturating material (Figure 3b-3d).

**Figure 4:**
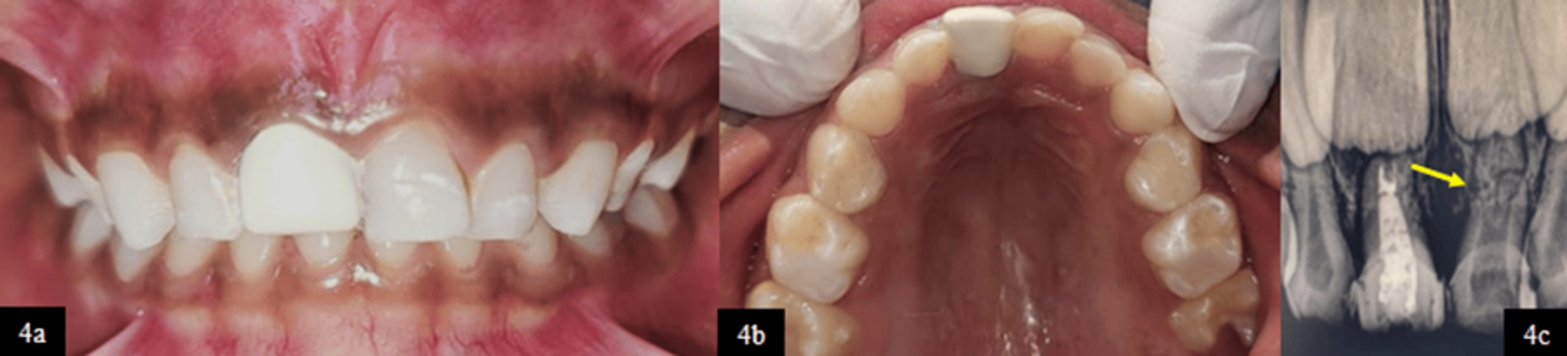
(4a) Frontal view at six-month follow-up showing BioFlx crown rehabilitation wrt 51. (4b) Maxillary occlusal view at six-month follow-up showing BioFlx crown rehabilitation wrt 51. (4c) RVG wrt 51 and 61 at six-month follow-up RVG, radiovisiography; wrt, with respect to

The six-month follow-up radiograph of tooth 61 revealed the stable alignment of the horizontal root fracture in the middle third of the root (Figure 4a-4c). There was no evidence of pathological root resorption and periapical radiolucency and signs of infection or further displacement. The fracture line remained well-demarcated, indicating favorable healing, with the permanent successor developing in an age-appropriate manner without any observable sequelae.

Case 3

**Figure 5:**
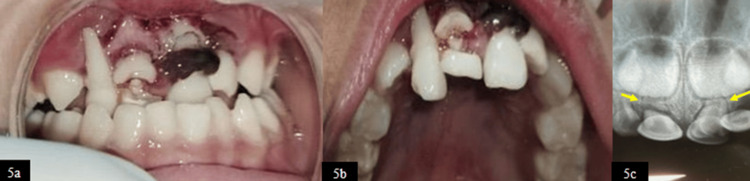
(5a) Frontal view showing extrusion wrt 51, 52, and 61. (5b) Maxillary occlusal view. (5c) Pre-operative RVG showing root fracture wrt 51 and 61 RVG, radiovisiography; wrt, with respect to

A five-year-old female patient presented with a history of trauma following a fall one day prior. Clinical examination revealed extrusive luxation exceeding 3 mm in both primary maxillary central incisors (teeth 51 and 61) and the left lateral incisor (tooth 62), with grade III mobility; a root fracture was associated with tooth 51, and complete root exposure was observed in the right lateral incisor (tooth 52) (Figure 5a, 5b). RVG assessment confirmed a horizontal root fracture at the middle third of the root of tooth 51 and at the apical third of the root of tooth 61 (Figure 5c).

**Figure 6:**
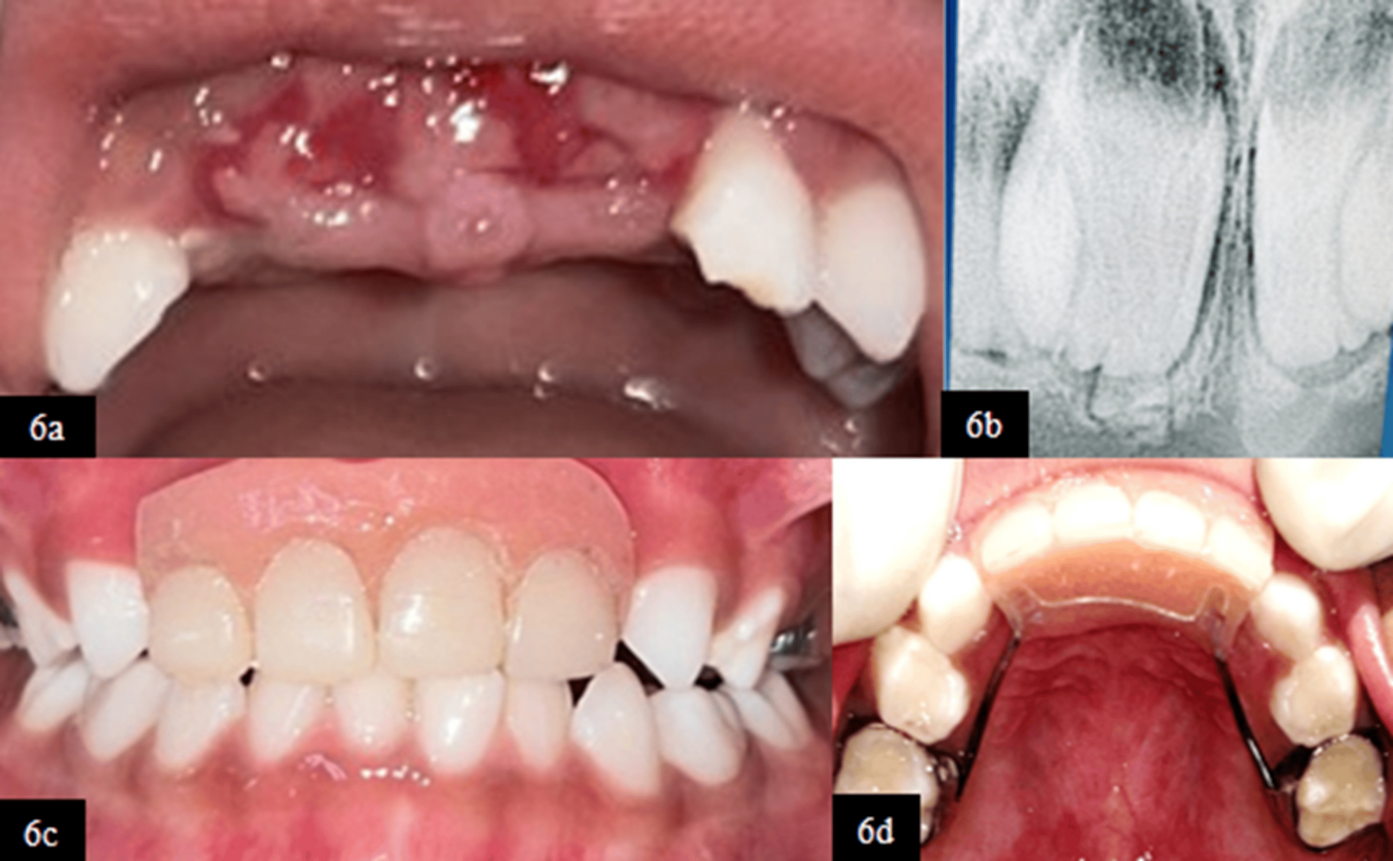
(6a) Frontal view after extraction wrt 51, 52, 61, and 62. (6b) Post-operative RVG wrt 51 and 61. (6c) Frontal view after the insertion of Groper’s appliance at two-month follow-up. (6d) Maxillary occlusal view after the insertion of Groper’s appliance at two-month follow-up RVG, radiovisiography; wrt, with respect to

Since the teeth were displaced palatally, excessively mobile, and interfered with occlusion, the extraction of primary maxillary central and lateral incisors was done under local anesthesia (2% lignocaine with 1:2,00,000 adrenaline) under active physical restraint as the child was highly uncooperative (Frankl Behavior Rating Scale {FBRS}: definitely negative) (Figure 6a, 6b). Successive follow-up appointments were scheduled to monitor the healing of the extraction site and gain the cooperation of the child using different behavior management techniques. Both clinical and radiographic evaluations at the two-month follow-up demonstrated the satisfactory healing of the extraction socket, following which a fixed Groper’s appliance was fabricated and inserted to provide esthetic and functional rehabilitation (Figure 6c, 6d).

**Figure 7:**
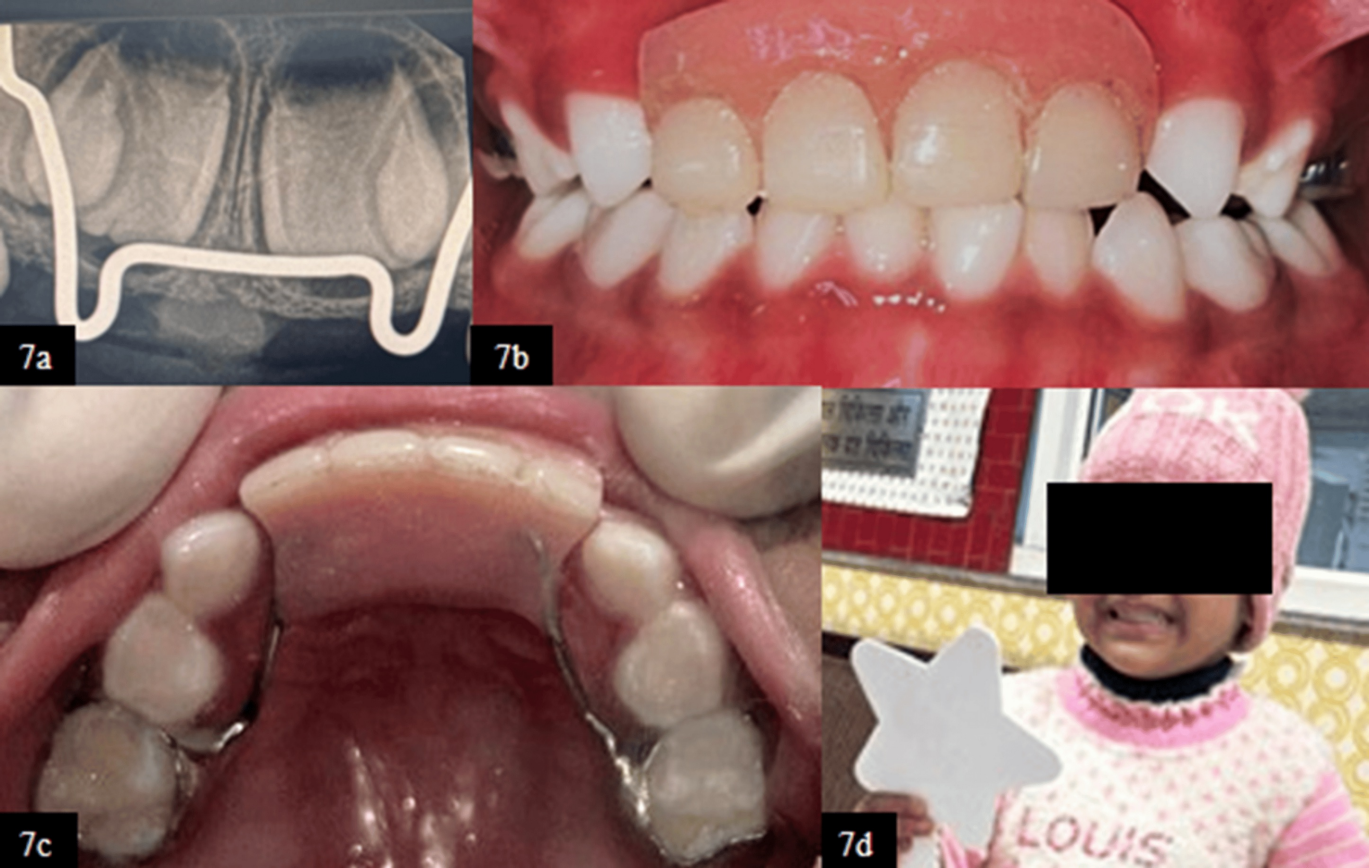
(7a) RVG wrt 51 and 61 at six-month follow-up. (7b) Frontal view at six-month follow-up. (7c) Maxillary occlusal view at six-month follow-up. (7d) Posttreatment extraoral photograph demonstrating patient satisfaction RVG, radiovisiography; wrt, with respect to

At six-month clinical follow-up, Groper’s appliance remained functional and well-adapted, with healthy surrounding soft tissues and satisfactory oral hygiene, while RVG revealed the developing permanent tooth buds of the maxillary central and lateral incisors in their normal anatomical positions, suggesting no disturbance in the eruption pathway (Figure 7a-7d).

## Discussion

Traumatic dental injuries in primary teeth are common incidents in early childhood, primarily due to the increased risk of falls and accidents during the developmental stages of motor coordination. The main objectives of the diagnosis and management of TDIs in children with primary dentition are pain relief, the prevention of possible damage to the developing permanent tooth bud, and minimizing the possibilities of sequelae. Effective behavior management is essential in pediatric TDI cases, as it facilitates accurate diagnosis and efficient treatment delivery and gives positive long-term dental attitudes and outcomes [13].

The management of root fractures in primary teeth presents a clinical challenge due to diagnostic complexities and therapeutic limitations, compounded by the young patient’s cooperation during treatment. Andreasen et al. (2002) concluded that prompt intervention in primary TDIs is essential to reduce the risk of complications [14]. The degree of mobility, displacement of the coronal fragment, and root status of the primary tooth influence the choice of treatment, which is based on the type of root fracture (Table 1). In cases of minimal displacement and mobility of the coronal fragment, it has been suggested that the fragment is left untreated. Conversely, significant mobility (grade III) or occlusal interference necessitates extraction when stabilization through splinting is not feasible [15,16].

**Table 1:** Classifications of horizontal root fractures based on various criteria Source: [10]

Fracture Line Location	Fracture Extent	Number of Fracture Lines	Coronal Fragment Position
Middle third (most common); apical third; coronal third	Partial; complete	Simple (single fracture); multiple; comminuted	Displaced; non-displaced

The close anatomical relationship between primary tooth apices and developing permanent tooth buds renders the latter highly susceptible to sequelae following trauma to the primary dentition (Table 2). Intrusion and avulsion injuries are most frequently associated with these long-term developmental disturbances [17,18].

**Table 2:** Sequelae in permanent teeth following trauma to primary dentition Source: [17,18]

Outcome Type	Prevalence (%)	Details
Enamel discoloration	53-61	Most common sequelae
Enamel hypoplasia	14.8-28.4	Defective or thin enamel
Root dilaceration	Up to 26.6	Especially after the intrusion
Eruption disturbances	10-18	Includes delay, ectopia, and impaction
Crown dilaceration	2-7	Less common
Tooth germ loss	<5	Rare but most severe

All patients had periapical radiographs recorded for diagnostic purposes. In both cases 1 and 2, although the patients presented with chief complaints involving different teeth, radiographs unexpectedly revealed more severe underlying traumatic root injuries. The coronal fragments in both cases demonstrated no mobility or displacement and were managed conservatively with routine follow-ups. In consideration of the patient’s young age in case 1, a two-year follow-up was essential to thoroughly monitor the healing process, detect any delayed complications, and ensure the normal physiological resorption of the fractured root fragment [7,18]. Similar outcomes were reported by Bruzda-Zwiech et al. (2018), who demonstrated the successful conservative management of a horizontally root-fractured primary incisor with no coronal fragment mobility through careful observation alone, emphasizing the role of long-term follow-up in achieving favorable healing [19]. Additionally, available evidence suggests that observational management results in positive outcomes in approximately 50%-54% of primary tooth root fractures over a 2-3-year follow-up period.

Due to the severity of the trauma and the mobility of the teeth in case 3, extraction was performed. A retrospective study by Kevci et al. (2023) reported that 42 out of 74 teeth with root fractures required extraction during the initial examination due to high coronal fragment mobility [18].

The premature loss of deciduous anterior teeth, particularly before the age of five, may lead to issues in phonetics and esthetics, associated with lower self-esteem in school-going children. The replacement of missing primary anterior teeth is often indicated not only for functional reasons but also to support the child’s psychological and emotional well-being by restoring natural esthetics. Groper’s appliance, introduced in 1984, was designed to address the esthetic and functional deficits associated with early anterior tooth loss. Its primary objective is to re-establish a natural appearance, thereby contributing to the child’s psychosocial development. However, the tendency for food debris accumulation remains a significant limitation, highlighting the need for thorough oral hygiene education and consistent compliance by both the child and their caregivers [20]. In the present case, a fixed Groper’s appliance was chosen over a removable option due to its superior retention, stability, and patient compliance, thereby minimizing the risk of appliance loss or damage and ensuring consistent functional and esthetic rehabilitation in a young patient.

The primary goal of pediatric dental care is to preserve primary teeth in the oral cavity until their natural exfoliation, as they play a crucial role in maintaining arch integrity, guiding eruption, and protecting the developing permanent tooth buds. This case series highlights the importance of the individualized, evidence-based management of root fractures in primary teeth, emphasizing the role of careful diagnosis, patient cooperation, and adherence to current guidelines, along with esthetic and functional rehabilitation to improve psychological well-being and oral health-related quality of life. Furthermore, it emphasizes that parents and caregivers often remain unaware of underlying root fractures, as demonstrated in case 1 and case 2, reinforcing the need to strongly advocate routine dental visits with counselling sessions and timely professional evaluation following any orofacial trauma, even in the absence of obvious symptoms. This case series also reflects the importance of long-term clinical and radiographic follow-up to monitor for potential developmental anomalies in the permanent dentition.

## Conclusions

Root fractures in the primary dentition, though less common, require timely diagnosis and appropriate management. Notably, such injuries are frequently accidental findings on radiographic examination, as clinical symptoms may be minimal or absent, highlighting the essential role of routine imaging in pediatric trauma assessment. The limited number of reported cases and variability in treatment approaches mark the need for studies with larger sample sizes to establish standardized treatment protocols and improve prognostic predictability. Therefore, this case series contributes to the existing literature by providing additional clinical insights into the diagnosis, management, and prognostic outcomes of traumatic dental injuries in the primary dentition.
